# The impact of vaccine hesitancy on psychological impairment among healthcare workers in a Total Worker Health^©^ approach

**DOI:** 10.3389/fpubh.2024.1447334

**Published:** 2024-09-12

**Authors:** Reparata Rosa Di Prinzio, Bianca Ceresi, Gabriele Arnesano, Alessia Dosi, Mariarita Maimone, Maria Eugenia Vacca, Maria Rosaria Vinci, Vincenzo Camisa, Annapaola Santoro, Massimiliano Raponi, Paola Tomao, Nicoletta Vonesch, Umberto Moscato, Salvatore Zaffina, Guendalina Dalmasso

**Affiliations:** ^1^Occupational Medicine Unit, Bambino Gesù Children's Hospital IRCCS, Rome, Italy; ^2^Alta Scuola di Economia e Management dei Sistemi Sanitari (ALTEMS), Università Cattolica del Sacro Cuore, Rome, Italy; ^3^Postgraduate School of Occupational Medicine, Università Cattolica del Sacro Cuore, Rome, Italy; ^4^Health Directorate, Bambino Gesù Children's Hospital IRCCS, Rome, Italy; ^5^Occupational and Environmental Medicine, Epidemiology and Hygiene Department, Italian Workers’ Compensation Authority (INAIL), Rome, Italy

**Keywords:** COVID-19, vaccine acceptance, mental health, flu, nurse, vaccine refusal

## Abstract

**Introduction:**

Vaccination practice is a well-known individual protective measure for biological risk in healthcare. During the COVID-19 pandemic vaccine hesitancy has grown among healthcare workers (HCWs). The study aims to investigate how vaccine hesitancy influences the psychological burden experienced by healthcare workers.

**Methods:**

This study aimed to explore attitudes of HCWs in acceptance or refusal of vaccinations related to the risk of psychological impairment (PI) and describe the associated occupational factors, during the seasonal flu/COVID-19 vaccination campaign of 2022–2023. 302 HCWs were enrolled in the study. A questionnaire was self-administered, including two scales on the risk of psychological impairment (Psychological Injury Risk Indicator, PIRI) and vaccine hesitancy (Adult Vaccine Hesitancy Scale, AVHS).

**Results:**

PIRI scores revealed that 29.8% of participants were at risk of PI. Differences in sex, age, occupational seniority, professional category, and night shifts were found between HCWs at risk of PI and those not at risk. Females registered a four-fold higher risk than males (85.6% vs. 14.4%, χ^2^ = 4.450, *p* < 0.05). Nurses were the highest risk category, followed by physicians and technicians (54.4% vs. 30.0% vs. 12.2%, χ^2^ = 14.463, *p* < 0.001). 41.7% of participants received the flu vaccination, and 98.9% received the COVID-19 vaccine. HCWs were prone to being vaccinated to protect patients and family members. Conversely, vaccine refusal was attributed to the perception of flu vaccines as not beneficial and COVID-19 contagion at low risk. The latter was more frequently reported for HCWs at risk of PI (16.7% vs. 4.7%, χ^2^ = 11.882, *p* = 0.001). Finally, hesitant HCWs were at higher risk of psychological impairment than others.

**Discussion:**

HCWs expressed vaccine acceptance considering their social role in the community as protectors. However, the underestimation of the risk of severity of COVID-19 was more relevant among HCWs at risk of PI than others. Psychological aspects need to be considered by healthcare providers when fighting vaccine hesitancy.

## 1 Introduction

Vaccination practice is a well-known individual protective measure for healthcare workers (HCWs), who are exposed to biological risk, to prevent nosocomial transmission of vaccine-preventable diseases (1–3). Since 2011 the NIOSH’s Total Worker Health^®^ approach has aimed to integrate the occupational safety and health protection with health promotion and disease prevention activities in the workplace. In this perspective, the occupational physician takes care of the global health status of workers, helping them improve their overall well-being and thus perform better in both working and extra-work situations (4).

The term “Vaccine hesitancy,” labelled among the top 10 threats to global health by the World Health Organization (WHO) in 2019 (5), represents an emerging behavior during the process of vaccination decision-making before the vaccination uptake (6). According to Working Group on Vaccine Hesitancy, vaccine hesitancy refers to delay in acceptance or refusal of vaccination despite the availability of vaccination services (7). Moreover, from an occupational viewpoint, costs related to lack of flu vaccination are well-known to the top management, raising awareness of the economic burden of vaccine hesitancy too (8).

Since the COVID-19 outbreak, HCWs have been facing extra psychological burdens including workplace violence (9) which requires specific psychological support (10). Psychological distress has been frequently expressed by several clinical manifestations [e.g., burnout, anxiety, depression, fear of transmitting infection, feeling of incompatibility, increased substance dependence, and post-traumatic stress disorder (PTSD)], especially shown in frontline HCWs (11). The interplay between psychological stressors and biological hazards among HCWs has been reported (12). Psychological determinants of vaccine hesitancy have been investigated as one of the most significant barriers to vaccination acceptance and uptake (13). Interestingly, PTSD has been found to have a mediating role between societal adaptation and vaccine worries in HCWs (14).

In addition, the COVID-19 pandemic has left negative effects on key antecedents of general vaccination affecting people’s general attitudes towards vaccination with a general increase in vaccine hesitancy (15). Furthermore, a new level of volatility around vaccine hesitation also includes the powerful impact of digital media platforms (16). Although HCWs reported a generally high acceptance of COVID-19 vaccination, with spikes of 86.20% in China and 91.50% among nurses in Italy (17), healthcare professionals have been surprisingly touched by the vaccine hesitancy phenomenon, resulting in a global emerging behavior in this professional category (18). The perception of receiving flu vaccination due to the professional duty that HCWs have in society has exacerbated the psychological burden of this professional category (19).

The aim of the study is to explore different attitudes in acceptance/refusal of flu and COVID-19 vaccinations in HCWs at risk or not of psychological impairment (PI). Moreover, we investigated the impact of vaccine hesitancy on the risk of PI.

## 2 Materials and methods

### 2.1 Study design, setting, and eligibility

An observational cross-sectional case–control study was conducted in a pediatric hospital in Rome. The enrollment of the participants took place at the Occupational Medicine Unit between October 2022 and January 2023 during the seasonal flu/COVID-19 vaccination campaign of 2022–2023. HCWs who accepted to receive the COVID-19 and/or seasonal flu vaccines were eligible to participate to the study by completing a self-administered questionnaire.

### 2.2 The questionnaire

A questionnaire was administered on paper by dedicated personnel of the Unit during health surveillance visits and vaccination sessions addressed to the hospital employees. Questionnaires with missing data were excluded from the study.

Sex and age were included among demographic variables, whereas professional categories included doctors, nurses and other healthcare jobs (e.g., technicians, biologists, pharmacists, sociosanitary operators). Work seniority, night shifts and agile work were also considered among occupational variables.

Psychological factors and vaccine hesitancy were explored too.

#### 2.2.1 Scale investigating the risk of psychological impairment

The Psychological Injury Risk Indicator (PIRI) is a standardized and validated questionnaire able to identify the tendency of psychological impairment (20). The Italian version was used (21). Given that Italian law requires HCWs to abstain from alcohol while at work, four of the five original subscales were considered, including sleep disorders (6 items), energy recovery (5 items), symptoms of post-traumatic stress syndrome (PTSD) (10 items), and chronic fatigue (5 items). Each item is scored on a seven-point Likert scale (0–6). The total score is calculated as the sum of the scores derived from each subscale; both the total and the subscale’s scores are standardized on a scale from 0 to 100. According to the original guidelines, overall scores higher than 25 indicate a potential risk of psychological injury (21). Based on the PIRI score, the population was divided into two groups, regarding HCWs at risk of psychological impairment (PI) and not at risk.

#### 2.2.2 Questionnaire on vaccine hesitancy

A 12-item questionnaire adapted from the Adult Vaccine Hesitancy Scale (AVHS) (22) was used to assess the attitude of HCWs towards COVID-19 and seasonal flu diseases and vaccine uptake. Four areas were explored, considering vaccine administration (4 items), reasons for vaccine acceptance (3 items), reasons for vaccine refusal (3 items) and knowledge (2 items). All areas explored seasonal flu, COVID-19 and flu/COVID-19 co-administration. Questions are reported in Supplementary Table S1.

### 2.3 Statistical analyses

Descriptive statistical analysis was conducted on demographics and occupational variables using mean and standard deviation for continuous variables and absolute and relative frequency for categorical variables. Since the normality test was not satisfied, the comparison between the two groups was performed using non-parametric tests (Mann–Whitney for continuous variables and χ^2^ test and Kruskal-Wallis test for categorical variables). Moreover, acceptance and refusal factors associated with the two vaccinations were compared in the two groups using χ^2^ test. *p* values <0.05 were considered statistically significant. Effect size was reported as r based on the Z statistic from the Mann–Whitney test for continuous variables, whereas Cramer’s V was used for dichotomous categorical variables. While we have included effect sizes and confidence intervals to enhance the interpretation of our findings, the study’s findings are subject to the limitations associated with multiple testing. Considering the overall population, a logistic regression model was then set up having the risk of PI as dependent variable and the significant resulted variables by univariate analyses as independent ones. In the logistic regression model development, variables statistically significant in the univariate analyses were selected for inclusion. This approach was employed to identify the factors most strongly associated with psychological impairment. Data were analyzed using IBM Statistics Package for Social Sciences (SPSS) (version 26.0).

## 3 Results

Out of 400 subjects examined during the sanitary surveillance, 302 HCWs were enrolled in the study (75.5%). They were mostly females (*n* = 235, 77.8%). The mean age was 39.8 ± 11.1.

### 3.1 Risk of psychological impairment

PIRI questionnaire total score showed an overall mean value below the cut-off (19.3 ± 16.0), indicating general good psychological health. On the contrary, the subscales related to energy recovery and sleep disturbance registered higher mean values than the considered cut-offs (33.5 ± 28.9 and 27.4 ± 22.4, respectively). Overall, 29.8% of the participants (*n* = 90) can be considered at risk of PI. This percentage increased to 45.7% for the sleep disturbance subscale and 51.3% for the energy recovery subscale. Moreover, over 30% of participants reported problems related to chronic fatigue and PTSD (Table 1).

**Table 1:** PIRI scores recorded in the study population.

	PIRI scores (mean ± SD)	HCWs at risk of PI
Total score	19.3 ± 16.0	90 (29.8%)
Sleep disturbance	27.4 ± 22.4	138 (45.7%)
Energy recovery	33.5 ± 28.9	155 (51.3%)
Chronic fatigue	21.3 ± 23.5	94 (31.1%)
Post-traumatic stress disorder	18.7 ± 21.4	92 (30.5%)

PI, psychological impairment; PIRI, Psychological Injury Risk Indicator.

By comparing workers at risk of PI to those not at risk, differences in sex, age, occupational seniority, professional category, and night shifts were highlighted. In detail, females registered a two-fold higher risk than males (85.6% vs. 14.4%, χ^2^(1, 302) = 4.450, *p* < 0.05). In addition, workers at risk of PI had higher age and seniority than workers not at risk (41.4 ± 10.5 vs. 38.2 ± 11.6, *p* < 0.01; 11.8 ± 11.7 vs. 7.2 ± 10.4, Mann Whitney’s U = 11491.00 and 12127.00 respectively, *p* < 0.001). Nurses were the professional category at higher risk, followed by physicians and technicians (54.4% vs. 30.0% vs. 12.2%, χ^2^(3, 302) = 14.463, *p* < 0.001). Night shifts represented a significant risk factor too (61.1% vs. 41.0%, χ^2^(1, 302)10.219, *p* = 0.001). On the contrary, agile work was not significantly different between the two groups. Given the small effect size statistics, significant variables should be considered with caution (Table 2).

**Table 2:** Comparison of demographic and occupational characteristics between the two groups.

Variables	HCWs at risk of PI (mean ± SD)	HCWs not at risk of PI (mean ± SD)	Mann Whitney’s U	r; 95%CI	*p* value
Age	41,4 ± 10,5	38,2 ± 11,6	11491.00	0.03; 37.85, 40.42	0.005
Work seniority	11.8 ± 11.7	7.2 ± 10.4	12127.00	0.05; 7.27, 9.77	<0.001

Variables	HCWs at risk of PI *n* (%)	HCWs not at risk of PI *n* (%)	Pearson’s χ^2^; OR	Cramers’ V; 95%CI	*p* value
Sex (female = 1 vs. male = 2)	77 (85.6%) vs. 13 (14.4%)	158 (74.5%) vs. 54 (25.5%)	4.450; 2.02	0.121; 1.73, 1.83	0.035
Job description			14.463	–	<0.001
Nurse vs. physician	49 (54.4%) vs. 27 (30.0%)	61 (28.8%) vs. 54 (25,5%)			
Nurse vs. technician	49 (54.4%) vs. 11 (12.2%)	61 (28.8%) vs. 55 (25.9%)			
Nurse vs. other	49 (54.4%) vs. 3 (3.3%)	61 (28.8%) vs. 42 (19.8%)			
Physician vs. technician	27 (30.0%) vs. 11 (12.2%)	54 (25.5%) vs. 55 (25.9%)			
Physician vs. other	27 (30.0%) vs. 3 (3.3%)	54 (25.5%) vs. 42 (19.8%)			
Technician vs. other	11 (12.2%) vs. 3 (3.3%)	55 (25.9%) vs. 42 (19.8%)			
Night shift (“no” = 0 vs.“yes” = 1)	55 (61.1%) vs. 35 (38.9%)	87 (41.0%) vs. 125 (59.0%)	10.219; 2.26	0.184; 0.41, 0.53	0.001
Agile work (“no” = 0 vs.“yes” = 1)	9 (10.0%) vs. 81 (90.0%)	26 (12.3%) vs. 186 (87.7%)	0.316; 0.80	0.032; 0.08, 0.15	0.575

95%CI: 95% Confidence Interval.

### 3.2 Vaccine administration

41.7% of participants got the flu vaccination and 31.5% had planned to get it. Most of the participants had been getting vaccinated for several years (66.8%), and 20.9% started after the COVID-19 outbreak; 12.3% had the first seasonal flu vaccination. The second COVID-19 booster dose was administered in 73.8% of subjects, followed by 16.2 and 8.9% of third and first booster doses, respectively. Co-administration was performed by half of the population, of which 20.9% had planned to get it, and 28.7% refused to get it.

Although not statistically significant, there is a trend of refusal of the flu vaccination and co-administration of flu/COVID-19 vaccines by HCWs at risk of PI. They also started to get flu vaccines since the pandemic outbreak (Table 3).

**Table 3:** Comparison of flu and COVID-19 vaccine administration prevalence between HCWs at risk of PI and not.

Items	HCWs at risk of PI *n* (%)	HCWs not at risk of PI *n* (%)	Pearson χ^2^	Cramers’ V; 95%CI	*p* value
Have you received the seasonal flu vaccination? (“yes” =1 vs. “not yet, but I will get it” =2 vs. “No, and I will not do it” =3)	33 (36.7%) vs. 31 (34.4%) vs. 26 (28.9%)	93 (43.9%) vs. 64 (30.2%) vs. 55 (25.9%)	1.051	0.067; 1.50, 1.68	0.305
How long have you been receiving seasonal flu vaccination? (“For a few years already” = 1 vs. “From the COVID-19 pandemic” =2 vs. “First time” =3)	48 (66.7%) vs. 16 (22.2%) vs. 8 (11.1%)	115 (66.9%) vs. 35 (20.3%) vs. 22 (12.8%)	0.003	0.029; 1.37, 1.56	0.954
Which COVID-19 dose did you receive? (“First booster” =1 vs. “Second booster” =2 vs. “Third booster” =3)	8 (8.9%) vs. 67 (74.4%) vs. 13 (14.4%)	19 (9.0%) vs. 156 (73.6%) vs. 36 (17.0%)	2.206	0.028; 2.04, 2.12	0.531
Did you perform flu/COVID-19 co-administration? (“yes” =1 vs. “No, but I am planning to do it” =2 vs. “No, and I am not planning to do it” =3)	39 (48.8%) vs. 17 (21,3%) vs. 24 (30.0%)	96 (51.1%) vs. 39 (20.7%) vs. 53 (28.2%)	0.130	0.022; 1.62, 1.84	0.719

95%CI: 95% Confidence Interval. PI: psychological impairment.

### 3.3 Vaccine acceptance, vaccine refusal, and knowledge

Overall, subjects referred to accept the vaccinations mostly to protect their patients and family members, and secondly to protect themselves; in addition, HCWs at risk of PI perceived a social meaning of COVID vaccination in the workplace context. Co-administration was accepted to address the severity of the diseases, according to official recommendations (Table 4A).

**Table 4:** Comparison of reasons for acceptance or refusal of vaccinations between HCWs at risk of PI and not.

Items	Answers (“no” = 0 vs. “yes” = 1)	HCWs at risk of PI *n* (%)	HCWs not at risk of PI *n* (%)	Pearson χ^2^; OR	Cramers’ V; 95%CI	*p* value
**a) Acceptance**						
If you intend to receive the seasonal flu vaccination or have already received it, what is/was the main reason?	To protect myself	31(34.4%)	89 (42.0%)	1.499; 0.726	0.070; 0.34, 0.45	0.222
	To protect my patients and family members	52 (57.8%)	125 (59.0%)	0.037; 0.952	0.011; 0.53, 0.64	0.849
	To conform to my departmental colleagues	0 (0.0%)	8 (3.8%)	3.489; 0.694	0.107; 0.01, 0.04	0.062
If you intend to receive the COVID-19 vaccination or have already received it, what is/was the main reason?	To protect myself	35 (38.9%)	100 (47.2%)	1.753; 0.713	0.076; 0.39, 0.50	0.186
	To protect my patients and family members	69 (76.7%)	157 (74.1%)	0.229; 0.028	0.028; 0.70, 0.80	0.633
	To conform to my departmental colleagues	3 (3.3%)	11 (5.2%)	0.492; 0.630	0.040; 0.02, 0.07	0.484
If you intend to receive flu/COVID-19 vaccinations co-administration or have already received it what is the main reason for accepting co-administration?	Reducing the number of visits to the vaccination center	13 (14.4%)	39 (18.4%)	0.692; 0.749	0.048; 0.13, 0.22	0.406
	Concern about the severity of both diseases and greater protection against the two viruses	23 (25.6%)	53 (25.0%)	0.010; 1.030	0.006; 0.20, 0.30	0.919
	Confidence in official recommendations	20 (22.2%)	47 (22.2%)	0.001; 1.003	0.001; 0.17, 0.27	0.992
**B) Refusal**						
If you are unwilling to vaccinate against seasonal flu, what is the main reason?	I do not consider the vaccine beneficial against the virus contagion	9 (10.0%)	11 (5.2%)	2.365; 2.03	0.088; 0.04, 0.09	0.124
	I do not consider the vaccine safe	2 (2.2%)	1 (0.5%)	1.968; 4.80	0.081; 0.00, 0.02	0.161
	Seasonal flu is not a serious disease	6 (6.7%)	15 (7.1%)	0.016; 0.94	0.007; 0.04, 0.10	0.899
	In recent seasons, flu has not been circulating and I believe it will be the same this year	2 (2.2%)	4 (1.9%)	0.037; 1.18	0.011; 0.00, 0.04	0.849
If you are unwilling to vaccinate against COVID-19, what is the main reason?	I do not consider the vaccine effective	3 (3.3%)	6 (2.8%)	0.055; 1.18	0.014; 0.01, 0.05	0.814
	I do not consider the vaccine safe	4 (4.4%)	3 (1.4%)	2.561; 3.24	0.92; 0.01, 0.04	0.110
	The Omicron variant of COVID-19 has no serious and severe effects	2 (2.2%)	4 (1.9%)	0.037; 1.18	0.011; 0.00, 0.04	0.849
	COVID-19 does not pose a risk to my health since I have already received an initial booster dose	15 (16.7%)	10 (4.7%)	11.882; 4.04	0.198; 0.05, 0.11	0.001
If you are unwilling to receive flu/COVID-19 vaccinations co-administration, what is the main reason for refusing co-administration?	Fear that the side effects may be more severe	17 (18.9%)	17 (18.9%)	0.669; 1.31	0.047; 0.12, 0.20	0.414
	I do not intend to receive the flu vaccination	21 (23.3%)	27 (12.7%)	5.308; 5.31	0.133; 0.12, 0.20	0.096
	I do not intend to receive the COVID-19 vaccination	10 (11.1%)	12 (5.7%)	2.779; 2.78	0.096; 0.04, 0.10	0.021
**C) Knowledge**						
Preferred sources	General practitioner/pharmacist/pediatrician	12 (13.3%)	25 (11.8%)	0.140; 1.151	0.021; 0.09, 0.16	0.421
	Social network/web	6 (6.7%)	12 (5.7%)	0.114; 1.190	0.019; 0.03, 0.09	0.459
	Printed or online newspapers/TV	4 (4.4%)	22 (10.4%)	2.826; 0.402	0.097; 0.05, 0.12	0.067
	Scientific journals/government websites (Ministry of Health/WHO/CDC)	47 (22.2%)	20 (22.2%)	–; 1.003	0.001; 0.17, 0.27	0.552
	Family/colleagues and/or friends	27 (30.0%)	65 (30.7%)	0.013; 0.969	0.007; 0.25, 0.36	0.512

95%CI: 95% Confidence Interval. OR: Odds Ratio; PI: psychological impairment.

The refusal of flu vaccination was attributed to the perception that it was not beneficial by HCWs at risk of PI, and because the seasonal flu was not considered a serious disease by HCWs not at risk of PI. The refusal of COVID-19 vaccination was mainly due to the perception of low risk of COVID-19 infection after having already received the full primary course plus a first booster dose; this idea was significantly more common among HCWs at risk of PI than others (Table 4B). In general, co-administration was refused because of the refusal of flu vaccination.

A range of 71.5–73.9% of participants in both groups reported that they knew about the possibility of receiving flu and COVID-19 vaccinations in the same session. The preferred sources were family/colleagues and/or friends followed by scientific journals/government websites (Ministry of Health/WHO/CDC) and General practitioner/pharmacist/pediatrician (Table 4C).

The multiple logistic regression showed that professional category, night shifts, and the underestimated risk of the severity of COVID-19 disease were determining factors of PI. Professional category, night shifts, and the belief that COVID-19 is not a severe risk were significant predictors of PI among HCWs. Specifically, being a physician or nurse increased the odds of PI, while working night shifts and underestimating the severity of COVID-19 decreased the odds of PI (Table 5).

**Table 5:** Multiple logistic regression.

	Pearson χ^2^	R^2^ of Cox e Snell	B	S.E.	Wald	gl	Sign.	Exp(B)
Model	54.671	0.166						
Work seniority			0.008	0.025	0.112	1	0.737	1.008
Sex			−0.634	0.388	2.667	1	0.102	0.530
Age			0.025	0.023	1.181	1	0.277	1.026
Professional categories					14.398	3	0.002	
Physicians			1.657	0.674	6.039	1	0.014	5.244
Nurses			1.927	0.692	7.747	1	0.005	6.871
Technicians			0.592	0.717	0.68	1	0.410	1.807
Night shift			−0.611	0.308	3.929	1	0.047	0.543
COVID is not a risk			1.336	0.534	6.262	1	0.012	0.263
No co-administration for COVID-19 vaccine refusal			−0.522	0.582	0.804	1	0.370	0.593
Constant			−1.252	1.174	1.138	1	0.286	0.286

## 4 Discussion

In literature, several factors influence vaccine administration, including personal factors and environmental factors. Regulations, professional duty, knowledge, individual perceptions of vulnerability, trust in the healthcare system, attitudes and past experiences are some of the identified determinants of vaccination (19, 22). Our findings showed that acceptance of flu and COVID-19 vaccinations reflected the social role of HCWs in protecting patients and family members before themselves. On the contrary, the refusal was mainly attributed to the perception of the flu vaccine as not beneficial and for COVID-19 the perceived low risk of contagion. The latter was more frequently reported for healthcare professionals at risk of psychological impairment, because the primary course plus a first booster dose should have been completed with full protection against the virus. Beyond occupational characteristics (e.g., professional category and night shift schedule), COVID-19 vaccine hesitancy impacted on the risk of PI among HCWs. According to the Total Worker Health^®^ approach (4), exploring the HCWs’ attitudes and perceptions of the biological risk in terms of vaccine hesitancy toward two of the most relevant vaccinations in recent decades is essential to raise individual and collective awareness of the updated evidence in the field.

Firstly, regarding the social role of HCWs, the desire to protect family and patients has been already documented in literature (23). In fact, HCWs performed a key role during the COVID-19 pandemic. Beyond their critical part in the frontline battle against the virus, HCWs bridged the gap between patients and healthcare institutions in a climate of social distancing and spread essential information about virus contamination management in the general population (24). HCWs’ social role is also identified in spreading good behaviors in the community, such as building confidence in vaccination decisions, as suggested by healthcare providers (25, 26). Attitudes and perceptions of HCWs toward vaccination practices are known to influence patient vaccine acceptance (25). The COVID-19 pandemic has raised the emphasis on trust or on the more sentimental aspects of decision-making surrounding health-protective behaviors like vaccination uptake (19). Perceived susceptibility, severity, and benefit of acting have been associated with COVID-19 vaccine acceptance among HCWs (25), with the highest rate for old male physicians (27, 28). Proper communication on these themes represents a strategic role in setting up successful immunological responses (29).

Secondly, in those who refused, a general perception of the seasonal flu vaccines as non-beneficial and the COVID-19 vaccine as not safe emerged. In the literature, a renewed ethical challenge has been outlined to solve the question of flu vaccine refusal among HCWs, which includes professional duty and ethics (deontology), self-determination and conscientious objection (30). The main reasons for refusing flu vaccination were lack of time, a feeling of invulnerability, conviction of not being at risk, of being too young or in good health, and misconceptions about vaccine efficacy (31). Previous evidence showed that suboptimal vaccination toward COVID-19 among HCWs was mainly due to concerns about vaccine safety, efficacy and potential side effects, because of the absence of educational campaigns, inaccurate risk perception, unknown or uncertain vaccination status, difficulties in accessing vaccination in the workplace, and equity-rooted challenges (26, 32). Low levels of education and awareness were described for those who used mass media, social media or Internet as the main source of information (17, 18, 33, 34). Beyond renewed cross-cultural differences related to age, gender, socioeconomic status, race, and social capital (35–38), occupational factors have been associated to vaccine hesitancy (39). In this respect, COVID-19 exposure, perceived risk, mandatory vaccination, and social pressure, altruism and collective responsibility were highlighted (39). A general nurses’ attitude of wait-and-see was reported (40), especially for females (17).

Thirdly, our findings showed a general underestimation of risk. Evidence from literature indicated that the low likelihood of contracting vaccine-preventable diseases and the perceived low severity of these diseases are the foremost reasons not to take the vaccines (41). Personal beliefs and misperceptions about the risk of contracting seasonal flu are known factors influencing the flu vaccine uptake (42). A pattern of underestimating the risk associated with flu and overestimating the risk of minor adverse reactions has been highlighted too (43). A review showed that although the cumulative increase in COVID-19 caseloads of countries over time, vaccination intention did not increase (44).

Finally, we found that COVID-19 hesitant HCWs were at higher risk of psychological impairment than others. In fact, emotional, cognitive, political, religious, moral, and cultural aspects may influence vaccination uptake (5). In literature, the role of psychological aspects on vaccine hesitancy has been widely reported. Low psychological well-being (e.g., anxiety, low confidence, low collective responsibility, low reward dependence, PTSD and trait reactance) influences the perception of COVID-19 vaccination acceptance and side effects (45–47). People suffering from pre-pandemic mental conditions were not prone to take up the vaccination (48). In addition, HCWs with PTSD symptoms and anxiety symptoms were more likely to be hesitant. Psychological barriers to vaccination have been summarized in the “5Cs model” comprising five relevant psychological antecedents of vaccination: confidence, complacency (risk perceptions), constraints (barriers), calculation (extent of information search), and collective responsibility (willingness to protect the community) (49). According to this model, HCWs’ vaccine decision-making process was mainly mined by the lack of trust in the COVID-19 vaccine, anti-science sentiment, adverse side effects, and situational risk assessment (13). Conspiracy theories have been reported too (18, 25, 50).

While counterintuitive, the effect of nightshift as a protective element could be linked to the fact that healthier HCWs do nightshift. In contrast, other HCWs with other comorbidities, including mental issues, are usually exempted. Besides, not considering COVID-19 as a risk could be linked to character traits that could be protective for PI.

In conclusion, our study strengthens the role of the psychological aspect of vaccine hesitant HCWs, which surely needs to be examined during the planning of future actions. However, some limitations may be outlined, including the cross-sectional design which does not allow to identify the causality between psychological impairment and vaccine hesitancy. Future longitudinal development in the upcoming vaccination campaigns could overcome this limitation. Moreover, considering the small effect size of the statistically significant evidence, practical implications should be taken with caution. Finally, the moderate fit of the logistic regression model (as indicated by the Cox and Snell R^2^ value of 0.166) suggests that while the model explains a portion of the variance, a significant amount remains unexplained.

## 5 Conclusion

Acceptance and refusal of flu and COVID-19 vaccinations are clearly modulated by several factors. Although persistent immunological memory after vaccination has been described as functional during breakthrough COVID-19 infections, vaccine hesitancy needs to be widely faced, especially when addressing HCWs (51). Enhancing the professional and social role of HCWs may be the keystone to address vaccine hesitancy for them and in the general population as well. In this regard, multicomponent interactive and context-specific interventions are fundamental in the tailored and clear communication approach to reach all people (13, 25, 52). HCWs’ psychological aspect needs to be considered in the process of strengthening the action of Public Health. From an occupational medicine perspective, systemic approaches may play an essential role in helping raise professional responsibilities among HCWs towards the general population (53).

## Data Availability

The original contributions presented in the study are included in the article/Supplementary material, further inquiries can be directed to the corresponding author.
