# Supplementary material for: The impact of vaccine hesitancy on psychological impairment among healthcare workers in a Total Worker Health© approach
Source: Front Public Health. 2024 Sep 12;12:1447334. doi: 10.3389/fpubh.2024.1447334 (PMC11425156; doi:10.3389/fpubh.2024.1447334)
Supplement: Supplementary file 1 [file Table_1.docx]

Supplementary Material

# Supplementary Data

**Table supplementary 1**. Questions for vaccine hesitancy adapted from Adult Vaccine Hesitancy Scale, AVHS (29).

| **Areas** | **Items** | **Possible answers*** |
| --- | --- | --- |
| Vaccine administration | Have you received the seasonal flu vaccination? | Yes |
|  |  | Not yet, but I will get it |
|  |  | No, and I will not do it |
|  | How long have you been receiving seasonal flu vaccination? | For a few years already |
|  |  | From the COVID-19 pandemic |
|  |  | First time |
|  | Which COVID-19 dose did you receive? | First booster (3rd dose) |
|  |  | Second booster (4th dose) |
|  |  | Third booster (5th dose) |
|  | Did you perform flu/COVID-19 co-administration? | Yes |
|  |  | No, but I am planning to do it |
|  |  | No, and I am not planning to do it |
| Reasons for vaccine acceptance | If you intend to receive the seasonal flu vaccination or have already received it, what is/was the main reason? | To protect myself |
|  |  | To protect my patients and family members |
|  |  | To conform to my departmental colleagues |
|  | If you intend to receive the COVID-19 vaccination or have already received it, what is/was the main reason? | To protect myself |
|  |  | To protect my patients and family members |
|  |  | To conform to my departmental colleagues |
|  | If you intend to receive flu/COVID-19 vaccinations co-administration or have already received it what is the main reason for accepting co-administration? | Reducing the number of visits to the vaccination center |
|  |  | Concern about the severity of both diseases and greater protection against the two viruses |
|  |  | Confidence in official recommendations |
| Reasons for vaccine refusal | If you are unwilling to vaccinate against seasonal flu, what is the main reason? | I do not consider the vaccine beneficial against the virus contagion |
|  |  | I do not consider the vaccine safe |
|  |  | Seasonal flu is not a serious disease |
|  |  | In recent seasons flu has not been circulating and I believe it will be the same this year |
|  | If you are unwilling to vaccinate against COVID-19, what is the main reason? | I do not consider the vaccine effective |
|  |  | I do not consider the vaccine safe |
|  |  | The Omicron variant of COVID-19 has no serious and severe effects |
|  |  | COVID-19 does not pose a risk to my health since I have already received an initial booster dose |
|  | If you are unwilling to receive flu/COVID-19 vaccinations co-administration, what is the main reason for refusing co-administration? | Fear that the side effects may be more severe |
|  |  | I do not intend to receive the flu vaccination |
|  |  | I do not intend to receive the COVID-19 vaccination |
|  | Did you know about the possibility of receiving flu and COVID-19 vaccinations in the same session? | Yes, I did |
|  |  | No, I did not |
|  | If yes, from where did you obtain this information? | General practitioner/pharmacist/pediatrician |
|  |  | Social network/web |
|  |  | Printed or online newspapers/TV |
|  |  | Scientific journals/government websites (Ministry of Health/WHO/CDC) |
|  |  | Family/colleagues and/or friends |
| *mark the answer most relevant to you (only one answer).  CDC: Centers for Disease Control and Prevention; WHO: World Health Organization. | | |
